# Understanding adjuvant endocrine therapy persistence in breast Cancer survivors

**DOI:** 10.1186/s12885-018-4644-7

**Published:** 2018-07-11

**Authors:** Leah K. Lambert, Lynda G. Balneaves, A. Fuchsia Howard, Stephen K. Chia, Carolyn C. Gotay

**Affiliations:** 1 School of Nursing, University of British Columbia, T201-2211 Wesbrook Mall, Vancouver, BC V6T 2B5 Canada; 2 College of Nursing, Rady Faculty of Health Sciences, University of Manitoba, 89 Curry Place, Helen Glass Centre for Nursing, Winnipeg, MB R3T 2N2 Canada; 3 British Columbia Cancer Agency, 600 W 10th Ave, Vancouver, BC V5Z 4E6 Canada; 4 School of Population and Public Health, University of British Columbia, V2206 East Mall, Vancouver, BC V6T 1Z3 Canada

**Keywords:** Adjuvant endocrine therapy, Medication persistence, Breast cancer, Cancer survivorship

## Abstract

**Background:**

Adjuvant endocrine therapy (AET) significantly decreases the risk of breast cancer recurrence and mortality. Notwithstanding the demonstrated efficacy of AET, 31–73% of breast cancer survivors do not persist with AET. The purpose of this study was to explore breast cancer survivors’ experiences and perspectives of persisting with AET and to identify the psychosocial and healthcare system factors that influence AET persistence.

**Methods:**

Informed by interpretive descriptive methodology and relational autonomy theory, individual interviews were conducted with 22 women diagnosed with early-stage breast cancer who had been prescribed AET. These participants also completed a demographic form and a survey that assessed their perceived risk of recurrence. Interviews were analysed using inductive thematic and constant comparative analysis to iteratively compare data and develop conceptualizations of the relationships among data. Descriptive statistics were used to summarize the quantitative data.

**Results:**

The personal, social, and structural factors found to influence AET persistence included AET side effects, perception of breast cancer recurrence risk, medication and necessity beliefs, social support, the patient-provider relationship, and the continuity and frequency of follow-up care. For most women, over time, the decision-making process around AET persistence became a balancing act between quality of life and quantity of life. The interplay between the personal, social, and structural factors was complex and the weight women placed on some factors over others influenced their AET persistence or non-persistence.

**Conclusion:**

Expanding our understanding of the factors affecting breast cancer survivors’ AET persistence from their perspective is the first step in developing efficacious, patient-centered interventions aimed at improving AET persistence. In order to improve AET persistence, enhanced symptom management is required, as well as the development of supportive care strategies that acknowledge the values and beliefs held by breast cancer survivors while reinforcing the benefits of AET, and addressing women’s reasons for non-persistence. Improved continuity of health care and patient-healthcare provider communication across oncology and primary care settings is also required. The development and evaluation of supportive care strategies that address the challenges associated with AET experienced by breast cancer survivors hold the potential to increase both women’s quality and quantity of life.

**Electronic supplementary material:**

The online version of this article (10.1186/s12885-018-4644-7) contains supplementary material, which is available to authorized users.

## Background

Breast cancer is the most common female cancer worldwide [1], and it is the second leading cause of cancer deaths in Canadian women [2]. Mortality, however, is declining due in part to effective treatments that include adjuvant endocrine therapy (AET) such as tamoxifen and aromatase inhibitors (AIs) [3]. In women with hormone-receptor positive (HR+) breast cancer, AET reduces the risk of recurrence by up to 50% [4]. Until recently, five years of AET was standard treatment for women with HR+ breast cancer. In 2014, the American Society of Clinical Oncology (ASCO) published guidelines that recommended AET be extended for up to 10 years in high risk women [5].

Persistence, defined as continuously taking AET for the prescribed treatment duration [6], has significant clinical relevance. Non-persistence has been associated with a reduced survival benefit for women who discontinue treatment early, with a significant increase in mortality (26%) for women who stop AET before the recommended five-year period [7]. Meta-analyses have shown the positive effect of long-term AET use, with five years of tamoxifen being significantly more efficacious in reducing breast cancer recurrence (rate ratio 0.82) and mortality (rate ratio 0.91) than only one to two years of AET [8]. Despite the demonstrated efficacy of AET, 31–73% of women with breast cancer are non-persistent in real-world settings [9].

To date, the literature has focused on identifying the demographic and clinical predictors of AET non-persistence. As a result, a gap exists in our understanding of why a substantial proportion of women do not persist with AET for the recommended treatment period. Studies have identified disease severity [10], comorbidities [11, 12], side effects [13], and type of breast surgery [12] as predictors of AET non-persistence. Younger (< 40 years) [12] and older (> 70 years) women [11, 14] are also at higher risk for non-persistence. Relatively few studies have examined the influence of personal (e.g., beliefs, values), social (e.g., social support, patient-healthcare provider (HCP) relationship), and structural factors in the healthcare system (e.g., continuity of care, access issues) [15–19]. Research suggests women with neutral or negative beliefs about the value of tamoxifen [10, 20, 21], body image concerns [22], limited social support [23], and dissatisfaction with their role in AET decisions [13] were more likely to discontinue treatment prematurely. A higher quality patient-HCP relationship [13], patient-centered communication [24], continuity of care [13], prescription drug coverage [25], and polypharmacy [21, 26, 27] were also positively associated with AET persistence. More recently, researchers have used qualitative methodology to explore women’s AET experiences and their resulting adherence and persistence decisions [15, 17–19]. What is unique about this study is the focus on the broader social and structural context and how these factors, along with personal factors, shape women’s AET experiences and persistence.

Increasing AET persistence has the potential to improve the efficacy of treatment and ultimately patient outcomes. While some progress has been made in understanding the personal, social, and structural factors associated with AET persistence, breast cancer survivors’ perspectives related to AET and persistence are notably missing from the literature. If effective, patient-centered strategies for targeting non-persistence are to be developed, it is essential we look beyond the identified demographic and clinical predictors, which provide an incomplete, and somewhat acontextual, understanding of AET non-persistence. Instead, qualitative inquiry that explores breast cancer survivors’ experiences and perspectives is needed to better articulate the multifaceted nature of AET persistence.

The aim of this study was to explore breast cancer survivors’ experiences and perspectives of AET use to describe how personal, social, and structural factors influence AET persistence.

## Methods

Drawing on intepretive description methodology [28] using the theoretical lens of relational autonomy, we qualitatively explored women’s experiences and perspectives related to AET persistence. Relational autonomy is an alternative interpretation of autonomy that considers the personal aspects of an individual’s life, while also acknowledging that social, political, and economic conditions can influence their decisions and behaviours [29]. The bulk of the research on AET persistence has predominantly focused on individual aspects (e.g., demographic, clinical characteristics) in isolation from the social and structural context that shape women’s AET decisions and behaviours. A relational autonomy lens was used to explore how the personal nature of AET-related decisions and the broader social and structural contexts influence breast cancer survivors’ AET experiences and resulting persistence. Specifically, the interview questions and the data analysis were guided by relational autonomy theory to explore factors such as beliefs, social support, relationships with HCPs, and access to and delivery of healthcare resources.

### Participant recruitment

**Fig. 1:**
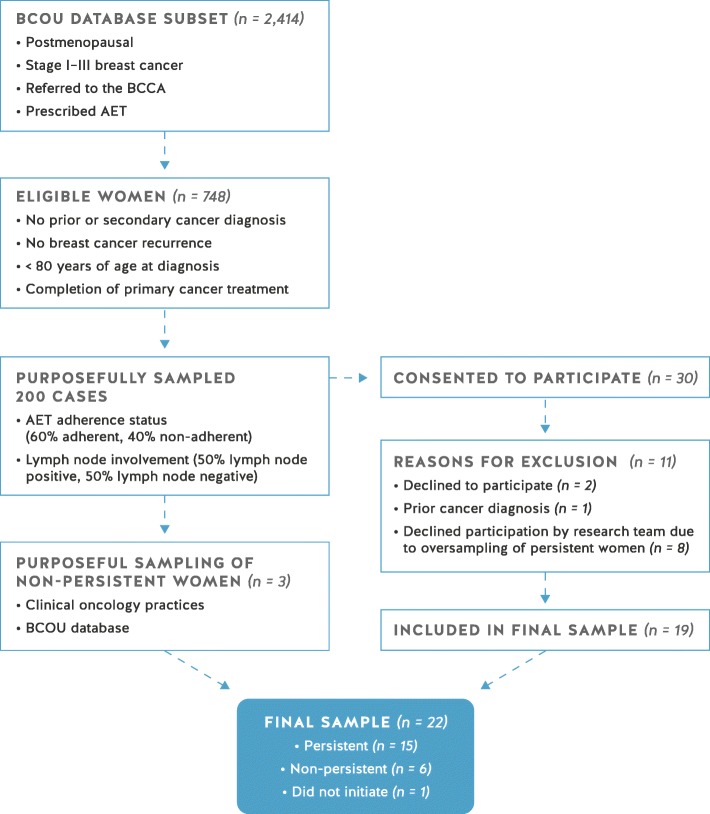
Sampling Diagram

Eligibility criteria included women diagnosed with HR+ stage I to III breast cancer, referred to the British Columbia Cancer Agency (BCCA) between January 2005 and August 2012, without a prior cancer diagnosis, recurrence of breast cancer, or secondary cancer diagnosis (excluding non-melanoma skin cancer), who had completed primary cancer treatment, were fluent in English, aged 18 to 79 years at diagnosis, and prescribed AET. The upper age limit of 79 years was chosen as an eligibility criterion to avoid contacting families of women who may have died since diagnosis. Upon approval from the appropriate institutional research ethics boards, 748 women who met eligibility criteria were selected from the BCCA’s Breast Cancer Outcomes Unit (BCOU) database subset (*n* = 2414), which had been generated for a previous study that found 40% of women with early-stage HR+ breast cancer were non-adherent to AET [30]. From this sample, a letter of invitation was mailed to 200 women who were purposefully selected from four randomized lists that represented diversity across adherence behaviour (adherent vs. non-adherent)^1^ as well as disease severity (lymph node positive vs. lymph node negative). A total of 30 women provided written consent to participate, of which four (13.3%) were non-persistent with AET, resulting in a 15% response rate. Following initial consent, two women declined to participate without providing a reason and one woman with a prior cancer diagnosis was excluded from the study. In addition, eight women persistent with AET, who had been waitlisted, were respectfully declined participation by the research team due to oversampling of persistent women in order to develop a sample that more closely represented the 40% non-adherence rate observed in the BCOU database [30]. Due to the difficulty in recruiting non-persistent women to the study, additional purposeful sampling was conducted through the BCOU database and convenience sampling was used to invite non-persistent women through select oncology practices. After several months of recruitment efforts, three additional non-persistent women consented to participate and were included in the final sample of 22 women (see Fig. 1).

### Data collection and analysis

The lead author (LKL) conducted semi-structured interviews with 22 women in person or by phone (see interview guide in Additional file 1), which were digitally recorded and transcribed verbatim. To address possible biases held by the researchers, the investigative team developed reflective memos regarding assumptions they held about the factors influencing women’s AET treatment decisions and behaviours prior to conducting the interviews. Field notes were kept to capture non-verbal behaviours occurring during the interviews and any related contextual information. The women completed a demographic form and one item, previously used by Andersen et al. (1999), which assessed their perceived risk of breast cancer recurrence on a 0 to 100 percentage scale, with 0 meaning there is no chance they will get breast cancer again and 100 meaning they most definitely will get breast cancer again [31]. Women received a $15 honorarium at the conclusion of the interview. Data was organized using NVivo™ software. Data collection and analysis occurred concurrently, with the preliminary analysis informing the development of new interview questions and shaping existing ones [32].

Inductive thematic analysis [33] was used to analyze the interviews. Transcripts were read and re-read line-by-line, with key passages highlighted and memos created to reflect important themes. Two members of the research team (LKL and LGB) reviewed several transcripts to confirm the proposed coding before the coding scheme was finalized. The analytic strategy of constant comparative analysis was used to iteratively compare data and develop conceptualizations of the relationships among data [32]. The analysis drew on relational autonomy theory by examining the interrelationship among themes and the personal, social, and structural factors influencing AET persistence. Memos were kept to track methodological and analytical decisions [34] and were reviewed during the analysis, along with the field notes. Descriptive statistics were used to summarize the quantitative data.

### Sample characteristics

Women classified as persistent (*n* = 15) were either currently taking AET at the time of interview or had recently completed the recommended five-year treatment. Within this group were some women who reported occasionally missing a dose or taking short medication breaks of less than two weeks. Women classified as non-persistent (*n* = 6) had discontinued AET before completing the recommended five-year treatment and one woman chose not to initiate AET. Sample characteristics and survey data are shown in Table 1.

**Table 1:** Sample Characteristics

Sample Characteristics *N* = 22	Frequency (%)
Age at diagnosis	
18–44 years	–
45–60 years	11 (50)
60–79 years	11 (50)
Lymph Node Status	
Positive	10 (45)
Negative	12 (55)
Hormone Status	
ER+	22 (100)
PR+	16 (73)
Her2+	2 (9)
Treatment History	
Surgery	22 (100)
Chemotherapy	9 (41)
Radiation	18 (82)
AET Use	
Tamoxifen only	7 (32)
AI only	5 (23)
Both tamoxifen and AI	9 (41)
Did not initiate	1 (5)
Persistence	
Non-persistent	6 (27)
Did not initiate	1 (5)
Persistent	15 (68)
Average Perceived Risk of Breast Cancer Recurrence (0–100%)	
Non-persistent/did not initiate	33%
Persistent	29%

## Results

**Fig. 2:**
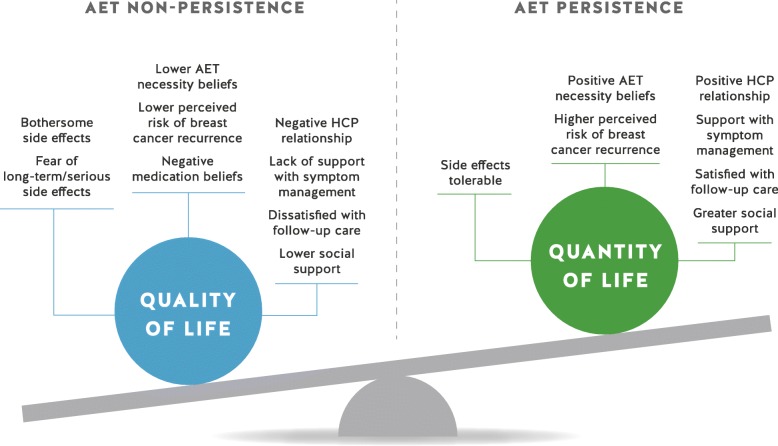
Balancing Act Between QOL and Quantity of Life in the Context of AET Persistence

For many breast cancer survivors, AET persistence became a balancing act between quality of life (QOL) and quantity of life (see Fig. 2) that was influenced by personal, social, and structural factors. These influencing factors and the interrelationships among them are described in detail below.

### Personal factors

Personal factors included experience of AET-related side effects and beliefs regarding perceived risk of recurrence, medication, and necessity of AET.

#### Side effects

AET-related side effects (see Burstein et al. [35] for a comprehensive list) had a profound impact on many women’s QOL, and were the primary reason for non-persistence in the sample. Many women did not frame their side effects as simply bothersome; rather, they used language such as “violent”, “excruciating”, and “intolerable” to describe their symptoms. They questioned whether the potential reduced risk of a breast cancer recurrence and increased survival benefit were worth persisting with AET given the severe negative physical and emotional impact on their daily lives.

I think the problem with breast cancer is that you’re not sick, but it [AET] makes you feel worse than you ever felt. The side effects are potentially worse than the disease. It’s like, ‘Why am I doing this?’ It’s bizarre. (AET non-persistent)

My joints and the cramping were sometimes unbearable. I would cry. When it would hit me at night, I would be sound asleep and it would jolt me out of my deep sleep. In the beginning I did not know how to deal with it. It affected me in my working environment and it affected me in my free time, and my family. (AET persistent)

Some of the symptoms, including hot flashes, vaginal dryness, weight gain, hair loss, and joint pain, were associated with old age and altered women’s sense of identity. As one woman shared: “It feels like you’re an 80-year old person. It’s hard to move around, to stand up, to get up out of the chair and do certain things” (AET persistent). The women’s social lives were also affected by the severity and unpredictability of AET-related side effects

I started to withdraw from social situations. I didn’t trust my body to co-operate. I missed out on quite a few things, because I was too afraid that [due to the diarrhea] I would have to run or, change my clothes or have a shower. And make a mess in public. Emotionally, it was devastating. (AET persistent after switching AETs)

A couple of women took AET medication breaks to lessen their symptom severity and allow them to attend important social events, such as vacations and weddings.

AET-related side effects also compromised some women’s ability to function in their occupational roles. These women missed work, were unable to maintain regular work schedules, or were prevented from returning to work. Several of these women chose not to disclose their struggle to employers or colleagues in an effort to avoid the stigma associated with breast cancer or appearing sick. Consequently, these women were not offered return to work programs or workplace accommodations (e.g. modified workloads) that might have helped them to cope with side effects. While not all women directly associated non-persistence with the negative impact of AET-related side effects on their careers, they did comment on how these work-related compromises had a substantial effect on their productivity, performance, and satisfaction with work.

Women’s ability to tolerate and self-manage side effects varied considerably over time and across the sample. For some, AET-related side effects improved after an initial adjustment period. For others, a change in medication offered some relief from intolerable side effects. To enable them to cope with the side effects and persist with AET, several women reframed their symptom experience and implemented mantras, such as “suck it up buttercup”. Other women made changes to their lifestyle to lessen the severity of side effects. There was a small group of women, however, who experienced intensified side effects as time went on, or found their symptoms became less tolerable, leading them to discontinue AET early.

#### Personal beliefs about recurrence and medications

How women perceived their risk of breast cancer recurrence differed across the sample and had a direct impact on their beliefs about the necessity of AET and tolerance of side effects, which ultimately influenced their persistence with AET. In most cases, a higher perceived risk of recurrence motivated women to remain persistent with AET. In contrast, women who perceived their risk to be low were more inclined to consider ending AET early: “Mine was so small and Stage I [breast cancer], so it wasn’t like a huge, life-threatening fact. So, I think not taking the pills would be better for me at the four-year mark” (AET non-persistent). In the interviews, women described their perceived risk of recurrence as being influenced by several factors, including disease severity, fear of recurrence, family history of breast cancer, previous illness experiences, anecdotal stories of breast cancer outcomes, and risk estimates provided by HCPs.

During the first few months of AET use, when the impact of the side effects became apparent, several women questioned their ability to continue with AET. A heightened perception of risk related to recurrence and a strong belief in the necessity of AET encouraged persistence during this initial treatment period. Increased perception of risk also occurred after follow-up consultations with HCPs, when new health concerns developed, and after the death of a family member or friend from cancer.

Being on these pills you start to forget what it's for. It's just like taking another pill, but when you get some of the symptoms, when they start to flare up, then it reminds you of actually what you're doing. And then you kind of have to go back into the fight mode again and say, ‘Okay, this isn't going to kill me.’ (AET persistent)

For non-persistent women, their perception of risk related to breast cancer recurrence decreased in the later stages of therapy. After the third year of AET, four (18%) women in our sample discontinued therapy. The perceived risk-benefit ratio appeared to shift for these women; they wanted their lives and bodies back, and to feel normal again.

Beliefs about the necessity of AET were largely influenced by how it was positioned in discussions with HCPs as an essential and expected step in the treatment trajectory. Hence, most women, be they persistent or non-persistent, described AET as a treatment option they could not refuse.

I have to say that my very first reaction on discovering I had the sort of breast cancer that needed more than surgery was, ‘I don’t want to take Tamoxifen’. I was prepared for everything else. But, I really, really was upset about the thought of taking Tamoxifen. I was devastated. I didn’t want to take something that was such a long-term thing. I knew I didn’t want to take it, but I knew I had to take it. (AET non-persistent)

For women who were AET persistent, they described holding positive beliefs about the medication, viewing it as essential to their health. AET was seen as a “security blanket”, an extra layer of protection in their fight against breast cancer and provided a sense of control over their disease: “It was a way to fight the disease and to make sure I didn’t get it back. I read about the side effects, but to me, it was all about winning the battle. I felt I was in control by doing everything in my power to fight this” (AET persistent).

Other women, however, feared overloading their body with “chemicals” and were concerned about the potentially serious and long-term adverse effects of AET. Furthermore, some women experienced difficulty reconciling the idea of taking a medication that had negative side effects with no immediate tangible benefits.

You want the good stuff that is helping your body, but if you don’t know for sure that it’s [AET] really helping your body, then why am I taking it? Do I really know that it’s benefiting me? And that’s probably why I wouldn’t take it again. Or, I wouldn’t do another five years. Because I haven’t seen the benefits yet. (AET persistent)

For some non-persistent women, these beliefs contributed to their decision to forgo or discontinue treatment early. In contrast, for those women who persisted with AET despite holding negative beliefs about medications, a heightened perception of risk of recurrence outweighed their concerns about taking a long-term medication: “It’s a drug in my body, doing things to me. There’s nothing good about doing it, but do I want to get cancer again? No. I’m more scared to get cancer than I am to go on the pill” (AET persistent).

### Social factors

#### Social support

Most women had a supportive social network, however, they perceived AET as a woman’s issue to be dealt with privately, shielding their family and friends from the challenges posed by AET. These women did not want breast cancer to continually impact their personal relationships and social interactions; instead, they wanted to move on with their lives, regain some sense of normalcy, and not be perceived as sick.

I want my life to be about other things. So, if people ask me how are you doing, I’m not shutting them out, but I don’t want to bring them into the full depth of it. I’ve been awake since three o’clock this morning ‘cause I woke up soaking wet and I’m grumpy and I don’t want to bring that to my friends and family all the time. So, I don’t talk about it as much with them. My husband knows. But I also don’t want our marriage to be just about that. (AET non-persistent)

In contrast, some women found the support from friends or family helped them persist with AET when side effects were bothersome and their commitment to AET waned, as did connections with fellow breast cancer survivors whose stories of overcoming difficulties and persisting with AET encouraged them to persevere with treatment. As well, anecdotal stories of survivors who took AET and survived had a powerful influence on women’s beliefs about the importance of AET, and consequently, their persistence.

#### HCP relationship

Close to half of the women (*n* = 10) continued to receive follow-up care from an oncologist throughout the course of AET. The remaining women (*n* = 12) were discharged to their family physician following primary treatment or after completing the first few years of AET. Women who perceived a positive relationship with their physicians and had a high level of trust and confidence in their recommendations about AET were more likely to persist. Further, women who perceived their physicians as empathic, responsive, accessible, and knowledgeable about AET were more inclined to discuss AET concerns in consults, seek help in managing side effects, and persist with AET. A breakdown in the patient-HCP relationship, however, damaged women’s trust in their physician, resulting in a perceived lack of support, poor symptom management, and for some women, influenced their decision to not persist with AET.

Oncologists were particularly influential in women’s decisions about AET persistence. A few women in our sample declined primary cancer treatment, yet agreed to take AET in an effort to preserve their relationship and access to follow-up care with their oncologist. Gaining permission from their oncologist was also key to women’s decisions to discontinue AET early, with some women sharing that if their physician had encouraged them, they would have tried to persist: “I said ‘I’ve decided to stop [taking AET], what do you think?’ And she [oncologist] shrugs and said ‘Fine’. If she had said ‘No, definitely not, I really don’t think you should stop’, I probably wouldn’t have” (AET non-persistent).

Some women had a lengthy history with their family physician, which led to a high level of trust. Other women did not perceive their family physician as having the specialized knowledge about breast cancer and AET required to provide adequate follow-up care. Their subsequent lack of confidence in their family physician prevented several women from seeking symptom management advice, resulting in unmet supportive care needs, which influenced some women’s decision not to persist with AET.

I wouldn’t go to the GP [family physician] because I don’t feel that they’re up on it [AET]. Well, I don’t feel mine is up on all that. They don’t have that knowledge. I think someone dealing with cancer, in the cancer setting, has more details on symptoms from one of those drugs. (AET persistent)

Disparities existed among women in terms of the support they received from HCPs with managing AET-related side effects. Women felt satisfied when their concerns were acknowledged and they were offered possible solutions. Some women were hesitant to ask their physicians about AET side effects because they feared their concerns would be met with resistance or apathy, or dismissed as being insignificant in comparison to the more severe side effects accompanying primary cancer treatment. As one woman shared:

He [oncologist] said you wouldn’t complain if you were on chemotherapy, given intravenously. You wouldn’t complain about the side effects. And I said, ‘No.’ And he said, ‘Well, look at it this way. You are taking a little bit of a chemo every day, and so you just have to learn to deal with it. (AET persistent)

### Structural factors

The transition from oncology to primary care was a key turning point for many women due to inequities in the provision of follow-up care. As mentioned, disparities existed regarding how breast cancer follow-up care was structured, with some women continuing to be followed by their oncologist for five years, while others were discharged earlier to a family physician. Some women experienced a lack of continuity of care when transitioning from oncology to primary care: “When I did go back to the family physician, I said ‘[my oncologist] dismissed me and it was up to you to keep track of me.’ And he [GP] looked at me and said ‘We don’t do that’” (AET non-persistent). In addition, differences existed in the care women received from oncologists versus family physicians in terms of the frequency and type of follow-up care. Dissatisfaction with the frequency and perceived quality of follow-up care provided by family physicians contributed to some women’s decision to stop AET early.

It [transition to primary care] was annoying because you know that means you’re really getting nothing. No follow up. Because you don’t get any follow up from a GP [family physician]. They say they don’t know anything about cancer, it’s too complicated. (AET non-persistent)

Conversely, women who continued to see an oncologist reported greater satisfaction with the provision of follow-up care as well as a sense of safety and confidence. The specific focus on breast cancer during follow-up visits with an oncologist meant that the importance of AET use and related symptom management issues were more frequently discussed than in follow-up visits with a family physician, when other health concerns took precedence.

Access to follow-up care was an additional issue for women residing in rural areas due to the limited number and availability of primary care providers. The inability to access HCPs with specialized knowledge of breast cancer and AET in a timely manner was disconcerting, especially when women’s worries felt immediate. One of the re-occurring issues most women struggled with was a perceived lack of time to discuss AET concerns with their physician.

The medical system is so overloaded and to deal with your GP [family physician] is difficult. They don’t give you much time. You wait two hours to see him [GP], and you get to talk to him for about two minutes. You have to talk kind of fast, and you never get what you wanted to say all out, because you have about two or three minutes. It’s not that conducive to getting a whole lot of help. (AET non-persistent)

Access to other HCPs, such as nurses and pharmacists with specialized knowledge of cancer and AET, provided a trusted, and often more accessible, resource for women. Inequities existed, however, in access to these supports. For instance, women participating in clinical trials had access to an interdisciplinary team who they relied heavily on to answer AET questions and provide help with managing side effects. Other women were not offered the same access to supportive resources. A lack of access to timely follow-up care meant some women felt abandoned during the survivorship period and were uncertain of how their breast cancer care would be provided, which in turn, influenced their decisions to stop AET early:

I wanted to be followed up. If they’re going to start fiddling with your hormone levels, they should be checking you every three months. There’s no checks and balances. If I had felt I was being followed and people knew what was happening to me, I would have felt much better. I felt totally alone. (AET non-persistent)

### Balancing quality and quantity of life

Most women reported that over time, the decision-making process around AET persistence became a difficult balancing act between QOL and quantity of life (see Fig. 2). The question, ‘What if?’ plagued women, who wondered if improved QOL and reclaiming a sense of normalcy was worth the increased risk of a breast cancer recurrence. For women who privileged quantity of life over QOL, positive beliefs about the necessity of AET and an acute perception of their risk of breast cancer recurrence tipped the scale towards persistence. For non-persistent women, the tipping point in their decision about AET was the relative weight they placed on QOL in relation to other factors, particularly side effects.

You’re counting the days and it becomes like you can’t wait for the end [of AET]. I don’t know what’s going to happen. It may come back and I’m going to die anyway. So, I’d rather have a good quality of life while I’m alive and not have side effects. (AET non-persistent)

While some persistent women were steadfast in their initial decision, others wavered throughout the course of AET. Several women reassessed their beliefs about the necessity of AET and their overall commitment when side effects intensified, concerns arose about potentially severe or late adverse effects of AET, perceived risk of recurrence decreased, a breakdown in the patient-HCP relationship occurred, and when they were dissatisfied with follow-up care.

The interrelationship among factors that influenced women to privilege either QOL or quantity of life, and to persist or not persist with AET, was complex and there was substantial variability in how women weighted the importance of various personal, social, or structural factors in their treatment decisions. For persistent women, social and structural factors, including support from family and friends and access to timely follow-up care with trusted HCPs who reinforced the value of AET, helped them persist when experiencing disruptive side effects and uncertainty about the necessity of AET. In contrast, non-persistent women struggled to continue with AET when their QOL was adversely affected, particularly in the face of insufficient support from their social networks and HCPs and beliefs that challenged the necessity and safety of AET.

## Discussion

The rate of AET persistence is low in breast cancer populations and tends to decrease over time [6, 14, 23, 36, 37]. Identification of patients at risk for non-persistence and the development of efficacious supportive care strategies are needed to improve women’s persistence. If the efficacy of AET demonstrated in clinical trials is to be realized, the patient-reported factors influencing persistence in real-world settings must be addressed. In this study, a relational autonomy lens was used to explore how personal, social, and structural factors shape women’s AET experiences, and how these factors interact to influence persistence throughout the AET trajectory. Our study found that breast cancer survivors’ decision to persist with AET was a balancing act between QOL and quantity of life and was informed by a complex interplay of factors. The relative weight women attributed to QOL and quantity of life at different points in the AET trajectory was grounded in their personal experience and how social and structural factors influenced the broader context of their AET decisions and behaviours.

Several quantitative studies have linked the presence and severity of AET-related side effects to non-persistence [13, 20–22, 38–41]. Our results echo these findings and further show the profound impact AET side effects can have on women’s QOL. Similar to previous qualitative studies [17–19, 42–45], we found that physical side effects, including weight gain, joint pain, and menopausal-like symptoms, greatly impacted women’s sense of self and body image and led some women to feel prematurely aged, which in turn influenced their decision to persist with AET. Given the significant value placed on youth and women’s physical appearance in Western society, it is not surprising that these side effects had an effect on AET persistence. This may be one area to address in future supportive care and lifestyle interventions offered to breast cancer survivors undergoing AET.

Research suggest that women’s self-determination, necessity beliefs, and their ability to tolerate side effects, can greatly influence AET persistence [15, 17–19]. In our study, tenacity and a strong belief in the importance of AET appeared to help persistent women cope with side effects through lifestyle modifications and committing to a positive mindset. This finding is similar to previous studies that found persistent women held more positive attitudes toward AET than non-persistent women [21, 46]. Translating these coping strategies into formal education resources (e.g., pamphlet, online resource, component of group education sessions) that could be shared with breast cancer survivors through oncology survivorship programs, primary care providers, and peer support groups could provide encouragement for women experiencing difficulty with AET. Given the limited pharmacological options available for treating AET-related side effects [47], interventions such as cognitive behavioural therapy, hypnosis, yoga, and relaxation strategies that have been effective in managing cancer-related symptoms might assist women to better cope with the difficulties of long-term AET [48].

In our study, we found that the importance women placed on influencing factors shifted over time. This finding is similar to the results of a recent study that examined the AET decision-making process and found concerns about AET can emerge at any point in the treatment trajectory, resulting in uncertainty and a subsequent reevaluation of AET decisions [49]. As noted in two recent qualitative studies, we also found that some women’s experience of AET-related side effects can improve over time [50, 51], however, the severity of side effects for other women continued or increased. Similar to Moon et al. (2017), we found the necessity beliefs related to AET for some women shifted throughout the treatment trajectory, leading them to question the important of AET. Unique to our study was the finding that breast cancer survivors’ perceived risk of recurrence can also shift over the course of therapy and influence women’s overall persistence.

Identifying modifiable factors, such as women’s perceived risk of recurrence and beliefs about the necessity of AET, provide potential avenues to explore the development of education and support strategies that promote AET persistence. Our findings suggest there are key milestones in the AET trajectory when women are at higher risk for non-persistence that could offer critical opportunities for intervention. For instance, studies have shown that AET non-persistence increases sharply during the first year of therapy [23, 38, 41, 46]. These results reflect the difficult adjustment period after initiating AET that cause some women to question their commitment to AET and may lead to non-persistence. It is important to note that while many women stop AET early in the treatment trajectory, the number of women who are non-persistent continues to increase over time [23, 52], as we saw with the women in our study who discontinued AET around the four-year mark. Furthermore, evidence from clinical trials of tamoxifen suggest that side effects continue to persist with longer durations of treatment [5], indicating the need for ongoing follow-up care throughout the entire course of AET, not only after initial onset. It is essential that HCPs assess AET persistence at each consultation, acknowledge women’s concerns, and seek to address reasons for non-persistence. In addition, as some women’s perception of risk decreases over time, the benefits of persisting with AET for the full treatment duration should be reinforced.

While side effects were the primary reason for AET non-persistence, there were women in our study who persevered despite experiencing severe side effects. This highlights the importance of identifying how social and structural contexts, in particular, the quality of the patient-HCP relationship and women’s trust in their physician, can either facilitate or hinder AET persistence. These findings are supported by previous research that suggests receiving adequate support from HCPs [13, 17–20, 50, 51, 53, 54], having frequent [55] and effective communication [24, 51], and trust in clinician advice [17, 43] might improve AET adherence and persistence.

Women reported greater satisfaction with care provided by oncologists compared to family physicians, which in turn facilitated persistence. Hadji et al. (2013) reported similar findings: women who received follow-up care from a specialist were more likely to be persistent with AET than women who received survivorship care within a general practice. As noted by Harrow et al. (2014) and Brett et al. (2018), we also found that women had concerns with the frequency and quality of follow-up care received from family physicians that influenced their decision to persist with AET. However, given the longevity of AET and the growing burden placed on oncology care programs, long-term breast cancer follow-up will need to increasingly occur in community settings. Alternative survivorship care models, such as nurse-led clinics [56], may be one strategy to address the supportive care needs of women undergoing AET, improve overall continuity of care, and increase persistence [57]. As a result, primary care providers will require increased knowledge of AET and related practice guidelines [58], awareness of the factors influencing persistence, and strategies for managing AET-related side effects to facilitate persistence and increase women’s QOL.

Standardized symptom management protocols for HCPs, telephone support lines, and peer support groups are just some of the possible strategies that might improve the experience of breast cancer survivors struggling with AET-related side effects. In addition, electronic resources such as evidence-based websites and online support groups might address the healthcare access issues and limited social support experienced by some women, particularly those living in rural and remote regions. Further to geographical differences, the discrepancy observed in our study regarding the provision of follow-up care (i.e., oncologist vs. family physician) also speaks to the potential inequities in how cancer survivorship care is delivered.

Our study results highlight a number of potential areas for further research. Identifying factors that influence AET persistence provide potential avenues to explore in the development of intervention strategies. Similarities between our results and the findings of recent research conducted across North America and Europe suggest that several factors found to influence AET persistence are not country specific, pointing toward the potential to develop universal intervention strategies that can be implemented across geographical regions [15, 17–19]. Given the recent guidelines recommending AET be taken for up to 10 years in certain populations [5], further research is needed to investigate how personal, social, and structural factors influence persistence in the context of extended therapy. Gaining HCPs’ perspectives will also be key to better understanding how the social and structural factors intersect to influence survivors’ AET persistence, and to inform the development of practical strategies for optimizing persistence. Most of the persistent women we interviewed experienced significant struggles related to AET side effects, indicating the importance of developing strategies to identify women facing AET-related challenges before they are lost to non-persistence.

### Limitations

The results of our study are limited by the small sample size comprised of predominately well-educated Caucasian women who reported a high socioeconomic status. There was some geographic variation within our sample that revealed the unique challenges experienced by women that reside outside urban settings. We acknowledge that the percentage of non-persistent women (32%) did not equal the 40% non-adherence rate observed in the database used to recruit our sample, which was due to difficulties in recruiting non-persistent women who had disengaged from the healthcare system and expressed limited interest in participating in research. Due to the large percentage of women who used both tamoxifen and an AI (41%) (see Table 1), we were not able to distinguish our findings between these two categories of AETs. While tamoxifen and AIs differ in their side effects profiles, there may be other distinguishing factors associated with different regimes that could have influenced women’s experience in persisting with AET. Our findings only reflect women’s perspectives and do not account for the experiences and perspectives of HCPs. Lastly, women may have experienced recall bias when reflecting on their experiences surrounding AET decisions and behaviours.

## Conclusion

The results of this study demonstrate that the personal, social, and structural factors influencing women’s AET persistence are complex and can shift over time. There is a growing body of evidence to support the impact of personal factors, such as side effects and women’s beliefs, on AET persistence. Further exploration of how the social and structural context in which AET decisions and behaviours are enacted is needed to guide the development of novel supportive care interventions. As well, it will be important to gain the perspectives of HCPs who support women undergoing AET to inform practical intervention strategies that can be implemented into routine clinical practice. Addressing women’s supportive care needs and ultimately AET persistence will help to ensure optimal survival outcomes for breast cancer survivors.

## Additional file

Additional file 1: Participant Interview Guide. Interview guide utilized in the study. (DOCX 21 kb)

## Abbreviations

AET: Adjuvant endocrine therapy
AI: Aromatase inhibitors
ASCO: American Society of Clinical Oncology
BCCA: British Columbia Cancer Agency
BCOU: Breast Cancer Outcomes Unit
HCP: Healthcare provider
HR+: Hormone-receptor positive
QOL: Quality of life

## Footnotes

1 The BCOU database used prescription refill data to assess breast cancer survivors AET adherence. After interviewing women, we found that AET persistence, not adherence, was the issue in our sample population and, therefore, women are referred to herein as persistent or non-persistent.
