# Supplementary material for: Understanding adjuvant endocrine therapy persistence in breast Cancer survivors
Source: BMC Cancer. 2018 Jul 11;18:732. doi: 10.1186/s12885-018-4644-7 (PMC6042363; doi:10.1186/s12885-018-4644-7)
Supplement: Supplementary file 1 — Participant Interview Guide. Interview guide utilized in the study. (DOCX 21 kb) [file 12885_2018_4644_MOESM1_ESM.docx]

**Participant Interview Guide**

1. What has it been like for you to take adjuvant endocrine therapy (AET) (aka hormone therapy)?
   1. What has been easy?
   2. What has been difficult?
   3. Are you dealing with any side effects from therapy? If so, could you say a bit more about that experience?
   4. Have you switched AET? (e.g., switching from tamoxifen to an AI) – Why? What was that like?
   5. What has motivated you to take AET?
2. What were you told about AET?
   1. What type of information were you given about AET (probe: studies, stories, etc.)
   2. Was this enough information? Too much? What was missing?
   3. Did you understand this information? If not, what are you uncertain about?
3. In your own words, can you tell me your understanding of how AET works?
4. What do you think are the pros and cons (benefits and risks) of taking AET?
5. How have you been followed up since taking AET?
6. What support have you received from your health care providers in taking AET?
7. Who has helped you make treatment decisions about AET?
   1. Probe health professional, family, friends etc.
8. What is your current thinking about your decision to take AET?
   1. Probe thoughts about quitting or continuing.
9. *If applicable* - Why did you decide to stop taking AET, switch AET or alter the dose of your AET?
   1. Did you consult with anyone in making the decision to stop/change/alter the dose of your AET?
   2. What would make you change your mind and decide to start taking AET again?
10. What has or would have been helpful in supporting you to continue with AET?
    1. If needed probe additional information, better communication with HCP, more information, educational resources, symptom management, reminders.
11. What strategies do you feel would be most helpful in supporting women in taking AET?
    1. What would these look like?
12. What messages do you think would be particularly powerful for women that have stopped early?
13. How does your decision (to continue or stop) AET fit with your other treatment and health care decisions?
14. There is beginning evidence on extending AET to 10 years, what are your thoughts about continuing for 5 more years?
    1. What kinds of things would you have to factor into that decision?
15. Is there anything else related to making a decision about AET you would like to share with me before we wrap up?
